# Exosomal circRNA: emerging insights into cancer progression and clinical application potential

**DOI:** 10.1186/s13045-023-01452-2

**Published:** 2023-06-26

**Authors:** Fan Zhang, Jiajia Jiang, Hui Qian, Yongmin Yan, Wenrong Xu

**Affiliations:** 1 Aoyang Institute of Cancer, Affiliated Aoyang Hospital of Jiangsu University, 279 Jingang Road, Zhangjiagang, Suzhou, 215600 Jiangsu People’s Republic of China; 2 Department of Laboratory Medicine, Wujin Hospital Affiliated with Jiangsu University, No. 2 North Yongning Road, Changzhou, 213017 Jiangsu People’s Republic of China; 3 Zhenjiang Key Laboratory of High Technology Research on sEVs Foundation and Transformation Application, School of Medicine, Jiangsu University, 301 Xuefu Road, Zhenjiang, 212013 Jiangsu People’s Republic of China

**Keywords:** Exosome, circRNA, Tumor immunity, Tumor metabolism, Biomarker, Cancer therapy

## Abstract

Exosomal circRNA serves a novel genetic information molecule, facilitating communication between tumor cells and microenvironmental cells, such as immune cells, fibroblasts, and other components, thereby regulating critical aspects of cancer progression including immune escape, tumor angiogenesis, metabolism, drug resistance, proliferation and metastasis. Interestingly, microenvironment cells have new findings in influencing tumor progression and immune escape mediated by the release of exosomal circRNA. Given the intrinsic stability, abundance, and broad distribution of exosomal circRNAs, they represent excellent diagnostic and prognostic biomarkers for liquid biopsy. Moreover, artificially synthesized circRNAs may open up new possibilities for cancer therapy, potentially bolstered by nanoparticles or plant exosome delivery strategies. In this review, we summarize the functions and underlying mechanisms of tumor cell and non-tumor cell-derived exosomal circRNAs in cancer progression, with a special focus on their roles in tumor immunity and metabolism. Finally, we examine the potential application of exosomal circRNAs as diagnostic biomarkers and therapeutic targets, highlighting their promise for clinical use.

## Introduction

Exosomes, the smallest component of extracellular vesicles (EVs), play a critical role in the tumor microenvironment (TME) by regulating its various components including tumor tissues, immune cells, stromal cells, endothelial cells and others. They carry biologically active molecules such as proteins, nucleic acids, lipids, and metabolites, which can have significant effects on the immune system, tumor metabolism, and drug resistance, thereby influencing the development of tumors [1–3]. TME-based components, particularly immune checkpoint inhibitors, have revolutionized cancer therapy [4, 5]. Exosomes play a crucial role in facilitating cell-to-cell communication between parent cells and recipient cells, thereby making a significant contribution to this field [6]. There is growing evidence that circRNAs, a type of non-coding RNA, play a crucial role in regulating cancer [7]. These circRNAs have unique advantages, such as their high stability and abundant content due to their circular structure, and the ability to be packaged into exosomes for intercellular transport. This leads to the formation of “exosomal circRNA,” which is emerging as an important biomarker and therapeutic target for cancer. However, the exact functions and mechanisms of exosomal circRNAs in tumor progression are not yet fully understood. Recent research shows that the expression of various exosomal circRNAs in the TME is often dysregulated. These exosomal circRNAs show great potential as diagnostic or prognostic biomarkers for cancer, due to their strong stability, long half-life, and resistance to degradation.

In this review, we focus on the roles and mechanisms of tumor and non-tumor cells-derived exosomal circRNAs in communicating with various cells in the TME, and their involvement in cancer-related processes such as immunity, angiogenesis, metabolism, drug resistance, proliferation and metastasis. Finally, we discuss recent advances in the use of exosomal circRNAs as cancer diagnostic biomarkers and new therapeutic strategies.

## Exosome and exosomal circRNAs

### Exosome biogenesis

Exosomes are composed of transmembrane lipid bilayers and various substances, including proteins, lipids, nucleic acids, and metabolites. These small vesicles are present in a range of body fluids, including blood, urine, thoracoabdominal fluid, cerebrospinal fluid, and saliva [8]. The biogenesis of exosomes occurs through the inward growth of the plasma membrane and the formation of multivesicular bodies (MVBs) [9]. Exosomes deliver proteins and nucleic acids to recipient cells through direct fusion with the cell membrane or various endocytic pathways, including phagocytosis, micropinocytosis, clathrin-dependent endocytosis, caveolin-mediated endocytosis, lipid raft-mediated endocytosis, and receptor-ligand interactions. Recent studies have found that many of the RNAs found in exosomes are different from those in their parent cells, which may be due to selective packaging of exosomes [10]. This highlights the complex and dynamic nature of exosome formation and contents.

###  Exosomal circRNA selectivity

CircRNAs are formed by reverse splicing of pre-mRNAs [11]. The biogenesis of circRNAs can be promoted by reverse repeat elements such as Alu and RNA-binding proteins such as QKI. These circRNAs have been found to accumulate in cells and have potential as biomarkers and therapeutic targets for various diseases, including cancer [12]. CircRNAs are abundant and stably expressed in exosomes [13], and some studies have shown that they can be packaged and function in exosomes. For example, circ_0088300 has been shown to be driven by KHDRBS3 and packaged into exosomes [14], and hnRNPA2B1 has been reported to package small RNA molecules, including circNEIL3 and circCCAR1, into exosomes [15–18]. However, the mechanism behind the selective packaging of specific circRNAs into exosomes is not yet clear and requires further investigation. Exosomal circRNAs have been shown to typically function as miRNA sponges or stabilizers [19, 20] and protein sponges or decoys [21]. They can also regulate parental gene transcription or serve as translation templates [22].

## Tumor-derived exosomal circRNAs in tumor progression

Mounting evidence highlights that certain exosomal circRNAs are expressed abnormally in cancer cells and tissues and can have an impact on tumor progression through mechanisms such as immune evasion, stimulation of angiogenesis, metabolic reprogramming, drug resistance, proliferation and metastasis.

### Tumor immunity

**Fig. 1:**
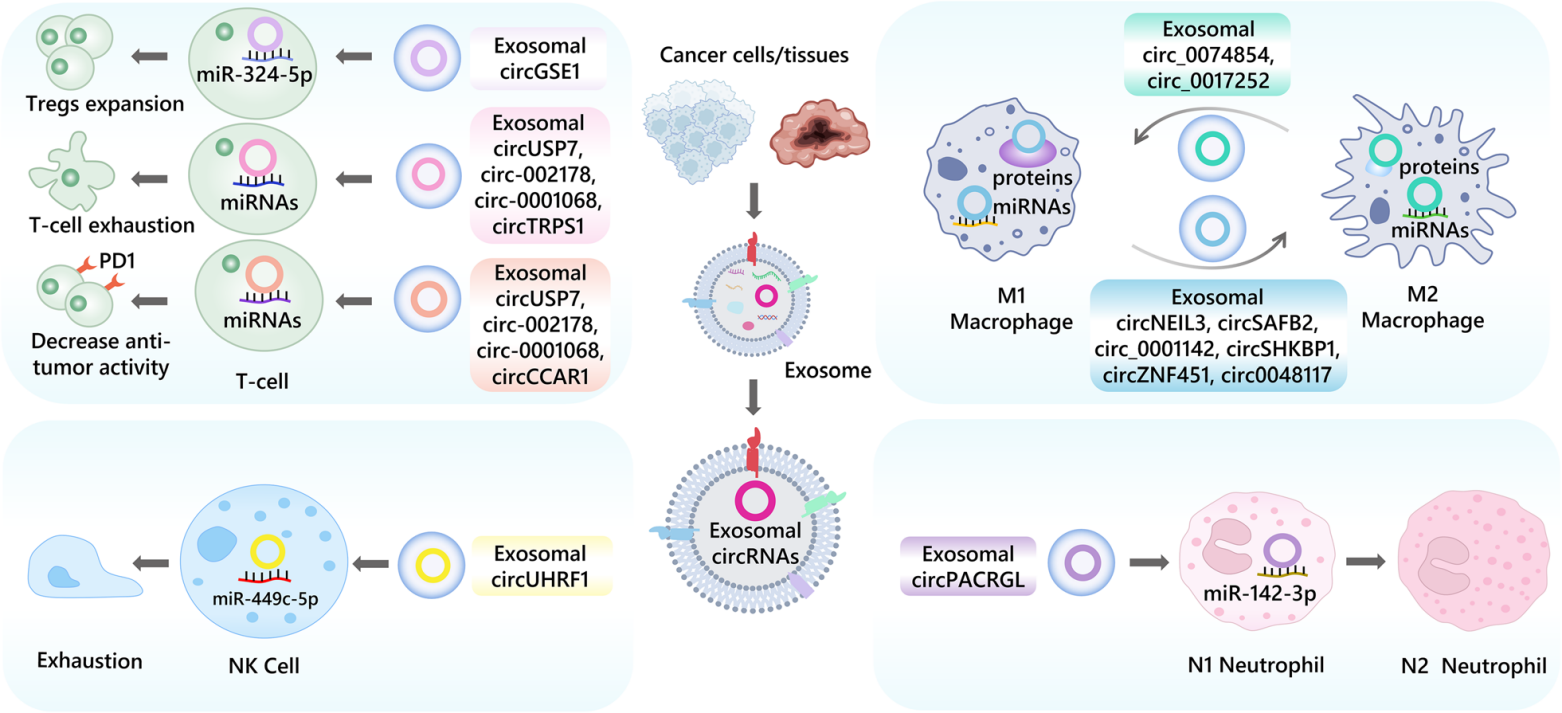
The role of tumor-derived exosomal circRNAs in immune cells, including T cell, macrophage, NK cell and neutrophil

The process of tumor immunity is a multifaceted and dynamic phenomenon that involves the participation of diverse immune cells and active molecules within the body. This intricate process encompasses various stages, including antigen presentation by Dendritic (DC) cells, T lymphocytes (T cell) activation, and anti-tumor activity of innate immune cells such as macrophages. However, tumor cells can evade the host immune response by means of several strategies, including the inhibition of tumor antigens, suppression of regulatory immune cells, and the secretion of immunosuppressive molecules [23]. In tumor tissues, communication between tumor cells and immune cells occurs through exosomes, which foster environment conducive to tumor growth [24]. Currently, there has been significant headway and progress in the exploration of exosomal circRNAs in regulating tumor immunity (Fig. 1 and Table 1).

**Table 1:** Tumor-derived exosomal circRNAs in tumor progression

Exosomal circRNAs	Parent cell	Target cell	Expression	Signaling pathway	Functions	References
*Tumor immunity*						
circCCAR1	Hepatocellular carcinoma cells	CD8 + T cell	↑	circCCAR1/PD1 deubiquitination	CD8 + T cell dysfunction, resistance to anti-PD1 immunotherapy	[18]
circUSP7	Non-small cell lung cancer cells	CD8 + T cell	↑	circUSP7/miR-934/SHP2	CD8 + T cell dysfunction, resistance to anti-PD1 immunotherapy	[26]
circRNA-002178	Lung adenocarcinoma cells	CD8 + T cell	↑	circRNA-002178/miR-34/PDL1/PD1	Immune escape, T-cell exhaustion	[29]
circTRPS1	Bladder cancer cells	CD8 + T cell	↑	circTRPS1/miR141-3p/GLS1	CD8 + T cell exhaustion, modulate intracellular ROS balance	[99]
circGSE1	Hepatocellular carcinoma cells	Tregs	↑	circGSE1/miR-324-5p/TGFBR1/Smad3	Immune escape, Tregs expansion	[28]
circ-0001068	Ovarian cancer cells	Jurkat T cell	↑	circ-0001068/miR-28-5p/PD1	Immune escape, induce PD1 expression	[49]
circSAFB2	Renal cell carcinoma cells	Macrophages	↑	circSAFB2/miR-620/JAK1/STAT3	Immune escape, promote M2 macrophage polarization	[31]
circNEIL3	Glioma cells	Macrophages	↑	circNEIL3/HECTD4/IGF2BP3	Promote M2 macrophage polarization	[17]
circ_0001142	Breast cancer cells	Macrophages	↑	circ_0001142/miR-361-3p/PIK3CB	Promote M2 macrophage polarization	[32]
circSHKBP1	Non-small cell lung cancer cells	Macrophages	↑	circSHKBP1/miR-1294/PKM2	Promote M2 macrophage polarization	[98]
circZNF451	Lung adenocarcinoma cells	Macrophages	↑	circZNF451/FXR1-ELF4-IRF4	Induce the pro-inflammatory phenotype of macrophages, CD8 + T cell dysfunction, resistance to anti-PD1 immunotherapy	[33]
circ0048117	Esophageal squamous cell carcinoma	Macrophages	↑	circ-0048117/miR-140/TLR4	Promote M2 macrophage polarization	[34]
circRNA cSERPINE2	Breast cancer cells	Macrophages	↑	cSERPINE2/MALT1/NF-κB/IL-6	Promotes IL-6 secretion, proliferation and invasion of BC cells and TAM recruitment	[37]
hsa_circ_0074854	Hepatocellular carcinoma cells	Macrophages	↓	hsa_circ_0074854/HUR	Suppress macrophage M2 polarization	[35]
hsa_circ_0017252	Gastric cancer cells	Macrophages	↓	hsa_circ_0017252/miR-17-5p/DUSP2	Suppress macrophage M2 polarization	[36]
circUHRF1	Hepatocellular carcinoma cells	NK cells	↑	circUHRF1/miR-449c-5p/TIM-3	Immune escape, NK cell dysfunction, resistance to anti-PD1 immunotherapy	[39]
circPACRGL	Colorectal cancer cells	Neutrophils	↑	circPACRGL/miR-142-3p/miR-506-3p-TGF-β1	Promote N2 neutrophil polarization	[46]
*Tumor angiogenesis*						
circSHKBP1	Gastric cancer cells	HUVECs	↑	circSHKBP1/miR-582-3p/HUR/VEGF and inhibit HSP90 degradation	Promote GC cell angiogenesis, proliferation, migration and invasion	[56]
circ29	Gastric cancer cells	HUVECs	↑	circ29/miR-29a/VEGF	Impair HUVEC proliferation, migration and tube formation	[57]
circ_0007334	Colorectal cancer cells	HUVECs	↑	circ_0007334/miR-577/KLF12	Promote cell viability, colony formation, migration, invasion and angiogenesis of CRC cells	[58]
circRNA-100338	Hepatocellular carcinoma cells	HUVECs	↑	circRNA-100338/VE-cadherin	Promote cell proliferation, angiogenesis, permeability, and vasculogenic mimicry (VM) formation ability of HUVEC	[59]
circKIF18A	Glioblastoma multiforme cells	Human brain microvessel endothelial cells	↑	circKIF18A/FOXC2/PI3K/AKT	Promote angiogenesis in GBM	[60]
circHIPK3	Breast cancer cells	Human endothelial cells	↑	circHIPK3/miR-124-3p/MTDH	Promote tube formation in ECs	[61]
circ-CCAC1	Cholangiocarcinoma cells	Endothelial monolayer cells	↑	circ-CCAC1/miR-514a-5p/YY1	Disrupt endothelial barrier integrity and induces angiogenesis	[62]
*Tumor metabolism*						
circ_0008928	CDDP-resistant cells	_	↑	circ_0008928/miR-488/HK2	Promote CDDP drug resistance, glycolysis metabolism, proliferation, migration, invasion in NSCLC	[67]
ciRS-122	Oxaliplatin-resistant cells	Oxaliplatin- sensitive cells	↑	ciRS-122/miR-122/PKM2	Promote glycolysis and transmits resistance to oxaliplatin	[71]
circNRIP1	Gastric cancer cells	Gastric cancer cells	↑	circNRIP1/miR-149-5p/AKT1/mTOR	Promote GC metastasis by altering metabolism and autophagy	[74]
circFBLIM1	Hepatocellular carcinoma cells	Hepatocellular carcinoma cells	↑	circFBLIM1/miR-338/LRP6	Promote HCC progression and glycolysis	[76]
circ-MEMO1	Non-small cell lung cancer cells	Non-small cell lung cancer cells	↑	circ-MEMO1/miR-101-3p/KRAS	Accelerate the proliferation, cell cycle progression, and glycolytic metabolism in NSCLC	[77]
circ-ZNF652	Hepatocellular carcinoma cells	Hepatocellular carcinoma cells	↑	circ-ZNF652/miR-29a-3p/GUCD1	Promote HCC progression, migration, invasion and glycolysis	[78]
circ_0072083	TMZ-resistant cells	TMZ-sensitive cells	↑	circ_0072083/miR-1252-5p/NANOG/ALKBH5	Promote TMZ resistance in glioma	[79]
circ93	Lung adenocarcinoma cells	Lung adenocarcinoma cells	↑	cir93/FABP3/AA	Essential for desensitizing LUAD cells to ferroptosis	[97]
*Tumor proliferation and metastasis*						
circIFT80	Colorectal cancer cells	Colorectal cancer cells	↑	circIFT80/miR-1236-3p/HOXB7	Promote CRC growth, proliferation, migration and invasion by activating EMT transcription factors	[100]
circSETDB1	Hypoxic cells	Normoxic cells	↑	circSETDB1/miR-7/Sp1	Promote aggressive growth and EMT in lung adenocarcinoma	[101]
circ-133	Hypoxic cells	Normoxic cells	↑	circ-133/miR-133a/GEF-H1/RhoA	Promote CRC cell migration	[102]
circRNA_PVT1	Cervical cancer cells	Cervical cancer cells	↑	circRNA_PVT1/miR-1286	Induce migration and invasion of cervical cancer cells, promote pulmonary metastasis	[103]
circ-0004277	Hepatocellular carcinoma cells	Surrounding normal cells	↑	circ-0004277/ZO-1	Promote HCC EMT progression	[104]
circWHSC1	Ovarian cancer cells	Peritoneal mesothelial cells	↑	circWHSC1/miR-145/MUC1 and circWHSC1/miR-1182/hTERT	Promote ovarian cancer occurrence and metastasis	[105]
circ-PDE8A	Pancreatic ductal adenocarcinoma cells	Pancreatic ductal adenocarcinoma cells	↑	circ-PDE8A/miR-338/MACC1/MET	Promote invasive growth of PDAC cells	[106]
hsa_circ_0000437	Gastric cancer cells	Human lymphatic endothelial cells	↑	hsa_circ_0000437/HSPA2-ERK	Promote lymph node metastasis, invasion, migration and tube formation	[110]
circ_0026611	Esophageal squamous carcinoma cells	Human lymphatic endothelial cells	↑	circ_0026611/NAA10/PROX1	Promote lymphangiogenesis	[111]
circZNF652	Glioblastoma cells	Glioblastoma cells	↑	circZNF652/miR-486-5p/SERPINE1	Promote GBM proliferation, migration and EMT malignant phenotypes	[112]
circPTGR1	Higher metastatic HCC cells	HCC cells with low or no metastatic potential	↑	circPTGR1/miR-449a/MET	Promote the increased migration and invasion capacity of HCC cells	[113]
circ-0072088	Hepatocellular carcinoma cells	Hepatocellular carcinoma cells	↑	circ-0072088/miR-375/MMP-16	Suppress HCC metastasis	[114]
circPUM1	Ovarian cancer cells	Peritoneum	↑	circPUM1/miR-615-5p/NF-kB and circPUM1/miR-6753-5p/MMP2	Promote ovarian cancer proliferation, migration and invasion	[115]
*Drug resistance*						
circATG4B	Oxaliplatin-resistant CRC cells	CRC cells	↑	circATG4B-222aa/TMED10/ATG4B	Increase autophagy followed by induction of chemoresistance	[116]
circ_0000337	CDDP-resistant cells	CDDP-sensitive cells	↑	circ_0000337/miR-377-3p/JAK2	Promote CDDP-resistant metastasis, cell proliferation, and metastasis in esophageal cancer	[117]
circVMP1	CDDP-resistant cells	CDDP-sensitive cells	↑	circVMP1/miR-524-5p-METTL3/SOX2	Promote CDDP-resistant metastasis in NSCLC	[118]
circWDR62	TMZ-resistant cells	TMZ-sensitive cells	↑	circWDR62/miR-370-3p/MGMT	Promote TMZ-resistant metastasis in gliomas, prognostic markers	[119]
circDNER	PTX-resistant cells	PTX-sensitive cells	↑	circDNER/miR-139-5p/ITGB8	Associated with PTX resistance in lung cancer, prognostic markers	[120]
circDLGAP4	Doxorubicin-resistant cells	Doxorubicin- sensitive cells	↑	circDLGAP4/miR-143/HK2	Promote doxorubicin resistance transmission, glycolysis, proliferation and invasion in NB	[121]
circZNF91	Hypoxic PC cells	Normoxic PC cells	↑	circZNF91/miR-23b-3p/SIRT1/HIF-1*α*	Enhance GEM resistance of PC cells	[122]

T lymphocytes are crucial cells in the adaptive immune response, serving as primary cells in the anti-tumor immune response [25]. In certain instances, exosomal circRNAs have exhibited regulatory effects on CD8 + T cells leading to dysfunction and reduced immune response. For example, the hepatocellular carcinoma (HCC) exosomal circCCAR1 can be delivered to CD8 + T cells and stabilize PD1 expression to promote CD8 + T cell dysfunction and anti-PD1 resistance in hepatocellular carcinoma [18]. In non-small cell lung cancer (NSCLC), exosomal circUSP7 inhibits the secretion of IFN-*γ*, TNF-*α* and tumor necrosis factors by CD8 + T cells dysfunction through miR-934/SHP2 axis, and reducing immune response [26]. Regulatory T cells (Tregs), a subset of T cells, can be recruited by tumor tissue and TME to promote and facilitate their proliferation, differentiation and secretion of immunosuppressive factors [27]. Exosomal circGSE1 induces Tregs amplification by targeting the miR-324-5p/TGFBR1/Smad3 axis, leading to promote hepatocellular carcinoma progression [28]. Interestingly, due to the ability of communication between tumor cells and immune cells, exosomal circRNA can modulate the role of T cells with direct or indirect pathway simultaneously. CircRNA-002178 could enhance PDL1 expression via sponging miR-34a in lung adenocarcinoma (LUAD) cells and also be delivered into T cells through tumor-secreted exosomes to promote PD1 expression via sequestering miR-28-5p, thereby inducing CD8 + T cell depletion and inhibiting their anti-tumor activity [29].

As far as innate immune, macrophages are often divided into two phenotypes: M1, which demonstrates anti-tumor properties, and M2, which is pro-tumor. Exosomal circRNAs are shown to regulate macrophage polarization and thus contribute to tumor progression and immune escape [30]. For instance, exosomal circSAFB2 facilitates M2 macrophage polarization in renal cell carcinoma (RCC), leading to increased immune escape and metastasis through miR-620/JAK1/STAT3 axis [31]. In glioma, after circNEIL3 packaged into exosomes by hnRNPA2B1 transmitted to infiltrated tumor-associated macrophages (TAMs), TAMs are endued immunosuppressive properties by stabilizing IGF2BP3 [17]. Exosomal circ_0001142 from breast cancer (BC) is released into macrophages, inducing M2 polarization and interfering with autophagy by targeting the miR-361-3p/PIK3CB pathway under the condition of endoplasmic reticulum (ER) stress [32]. Similarly, exosomal circZNF451 in LUAD promotes ubiquitination of FXR1 and activates the ELF4-IRF4 pathway to induce macrophage polarization and CD8 + T cell dysfunction [33], exosomal circ0048117 under hypoxic conditions can be delivered from esophageal squamous cell carcinoma (ESCC) cells to macrophages and cause M2-type polarization [34]. In contrast, low levels of hsa_circ_0074854 inhibit migration and invasion of HCC cells by interacting with human antigen R (HuR), while inhibiting M2 polarization [35]. Exosomal hsa_circ_0017252 secreted by gastric cancer (GC) cells also effectively inhibits M2 polarization of macrophages and GC invasion by sponging miR-17-5p [36]. It is noteworthy that exosomal circRNA cSERPINE2 in BC cells can cause high IL-6 secretion in TAM through the MALT1-NF-κB-IL-6 axis, while increased IL-6 could positively feedback to enhance ELF4A3 and CCL2 levels to promote cSERPINE2 synthesis and recruitment of TAMs [37].

Natural killer (NK) cells are an essential subpopulation of innate lymphocytes that serve a crucial function in cancer defense [38]. A study has demonstrated that exosomal circUHRF1 is highly expressed in caner tissues and plasma exosome of patients with HCC, while the number of NK cells in peripheral blood is decreased. It induces NK cells exhaustion and reduced secretion of IFN-*γ* and TNF-*α* through the degradation of miR-449c-5p and upregulation of TIM-3 expression, which then inhibits HCC progression [39]. Although limited research exists regarding the role of exosomal circRNA in NK cells, this study highlights the impact that exosomal circRNA has on the outcome of cancer, such as HCC, through its influence on NK cell function and exhaustion (Fig. 1).

Neutrophils serve a crucial role in tumor immunosuppression and can contribute to the tumor progression [40]. Tumor-associated neutrophils (TANs) have been identified as either an N1 (anti-tumor) or N2 (pro-tumor) phenotype [41]. Notably, exosomes have been observed to promote the polarization of neutrophils to N2 phenotype and facilitate the migration of neutrophils [42]. Exosome can also mediate the communication between tumor cells and neutrophils impact tumor progression [43–45]. For instance, exosomal circPACRGL derived from colorectal cancer (CRC) influences the progression of CRC through the miR-142-3p/miR-506-3p-TGF-β1 axis, while also regulating the differentiation of N1-N2 neutrophils. In contrast, the proportion of N2 neutrophils is significantly decreased in circPACRGL knockdown cells [46] (Fig. 1).

Immune checkpoints are critical mechanisms that allow tumors to escape immune attack, mediated through the expression of immune checkpoint proteins such as CTLA4, PD1/PDL1, and TIM-3 [47]. Tumor cells can achieve immune escape by increasing the expression of immune checkpoint proteins such as PD1 and PDL1 [48]. Exosomal circRNAs have been confirmed to play a role in the interaction between tumor cells and immune cells, affecting the expression of immune checkpoint proteins and regulating the anti-tumor activity of immune cells (Fig. 1). For example, circ-0001068 in ovarian cancer can be delivered to T cells via exosomes and targets miR-28-5p as well as induces the expression of PD1, causing immune escape of T cells [49]. Exosomal circCCAR1 stabilizes PD1 expression in CD8 + T cells, causing anti-PD1 resistance in HCC [18].

Collectively, based on the characters of abundance and intercellular communication, exosomal circRNAs involve in remodeling the TME directly or indirectly, and in turn regardless the immune cells or immune checkpoint molecules are both almost affected. In light of these findings, it is critical to explore new targets for immunotherapy based on exosomal circRNAs. However, much remains unknown about the relations between exosomal circRNAs and the immune system, particularly with regard to interactions with various immune cells. Further investigation is necessary to understand the impact of exosomal circRNAs on immune function in the context of cancer.

### Tumor angiogenesis

**Fig. 2:**
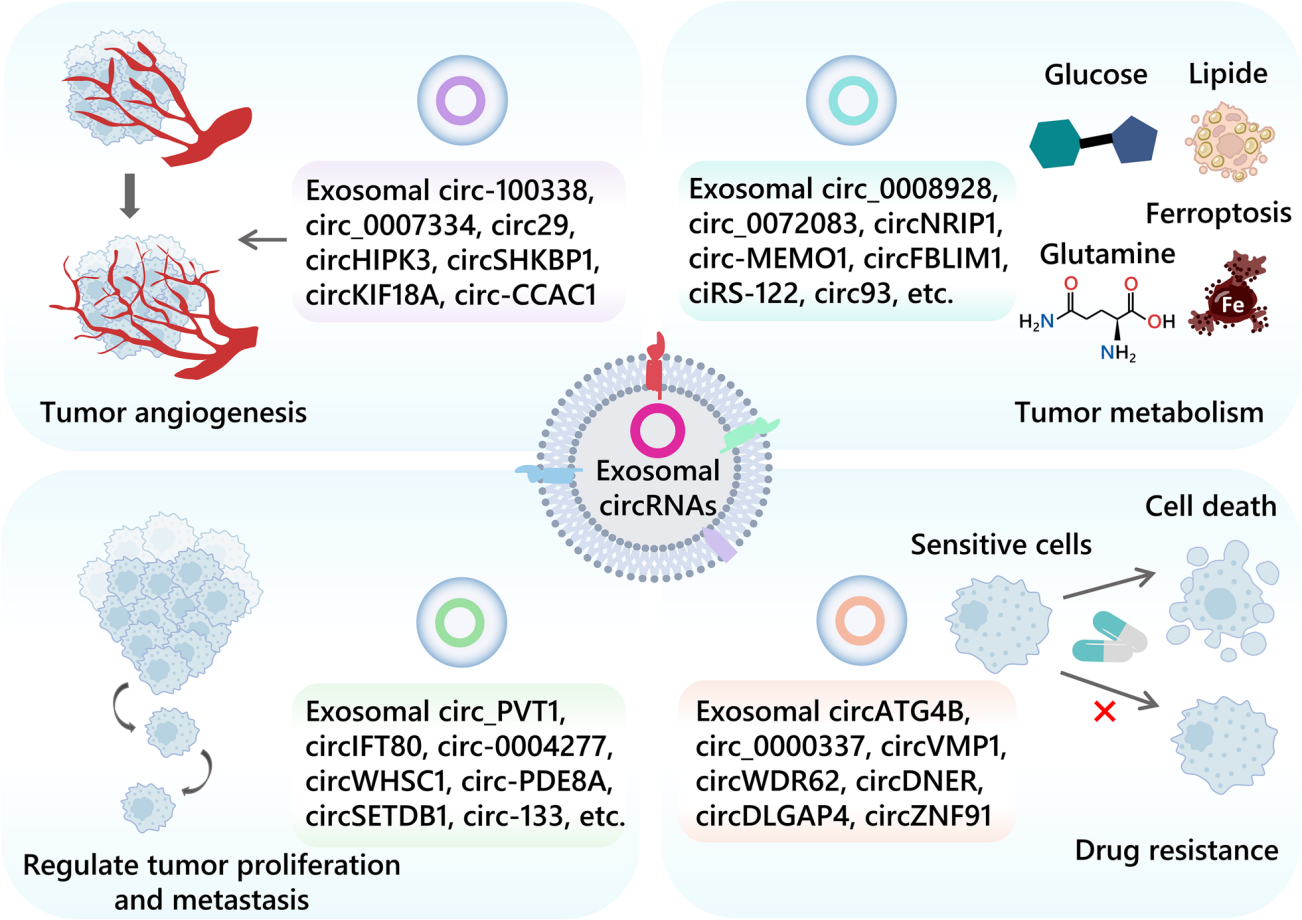
The roles of tumor-derived exosomal circRNAs in the tumor angiogenesis, metabolism, migration, metastasis and drug resistance

Angiogenesis, the formation of new blood vessels, is a key feature of cancer and a crucial pathway in tumor development [50]. The activation and production of multiple angiogenic factors in TME and the rapid growth of tumor cells contribute to the development of vascular networks [51–54]. Vascular endothelial growth factor (VEGF) is a commonly produced angiogenic factor that plays an important role in physiological and pathological angiogenesis [55]. Xie et al. have demonstrated that circSHKBP1 is up-regulated in serum exosomes of gastric cancer patients, and it is correlated with vascular infiltration and TNM stage (Fig. 2 and Table 1). Mechanistically, circSHKBP1 promotes GC progression by upregulating HUR and VEGF through sponge miR-582-3p and decoy HSP90 by competing with STUB [56]. Moreover, exosomal circ29 [57], circ_0007334 [58] and circRNA-100338 [59] are released by tumor cells into human umbilical vein endothelial cell (HUVEC) and promote angiogenesis. Furthermore, glioblastoma-associated microglia-derived exosomal circKIF18A regulates nuclear translocation in human brain microvascular endothelial cells (hBMECs) and promotes angiogenesis in glioblastoma multiforme (GBM) [60]. In addition, many other exosomal circRNAs have been shown to promote angiogenesis [61, 62].

These findings indicate that exosomal circRNAs have the ability to boost the process of angiogenesis, thereby influencing the progression and inflation of tumor, and making them potential targets for anti-angiogenic therapies.

### Tumor metabolism

Metabolism reprogramming and dysregulation accompany with tumor progression and provide the essential source to the tumor cell growth and division [63]. In a comparative study of HCC gene mutations, a cluster of metabolic disease-associated HCC was determined, which was intimately linked with metabolic risk factors such as diabetes and obesity [64]. In fact, there are many cancers associated with the metabolic disease and exosomal circRNA involves in the cancer metabolism.

In the hypoxic TME, malignant tumors usually rely on aerobic glycolysis to produce ATP, leading to resistance to chemotherapy and rapid growth, which is commonly referred to as the Warburg effect. Tumor tissue highly expresses HK2, which acts as the first rate-limiting enzyme of the glycolytic pathway to drive growth. An HK2 inhibitor with a glucose-like structure, 2-deoxy-D-glucose (2-DG), competes with glucose for hexokinase to inhibit glycolysis and synergistically enhances the anticancer effect with sorafenib [65, 66]. Circ_0008928 highly expressed in serum exosomes and cells of cisplatin-resistant NSCLC patients can sponge miR-488 and upregulate HK2 to promote glycolysis and tumor progression. The combination of si-circ_0008928 and 2-DG may lead to a better therapeutic outcome for cisplatin-resistant NSCLC [67]. Pyruvate kinase type M2 (PKM2), which is highly expressed in cancer cells, catalyzes the final reaction in glycolysis by transferring phosphoenolpyruvate (PEP) to ADP to produce ATP and pyruvate [68], and also strongly associated with chemoresistance in many types of cancer [69, 70]. Exosomal ciRS-122 from oxaliplatin-resistant CRC cells can be delivered to sensitive cells and target miR-122 to increase PKM2 expression levels, further promoting glycolysis and drug resistance (Fig. 2 and Table 1) [71]. Lactate dehydrogenase A (LDHA) is another important player in the Warburg effect. Phosphorylation of the LDHA residue (Tyr142) promotes the conversion of NADH to NAD + and the generation of pyruvate and lactate, which are associated with multiple cancer-promoting mechanisms. CircFOXK2 can encode FOXK2-142aa to promote LDHA phosphorylation while regulating the miR-484/Fis1 pathway inducing mitochondrial division, and ultimately activating aerobic glycolysis and lung metastasis in HCC. This shows the important value of circRNA-translated proteins in cancer metabolism [72]. The AKT/mTOR pathway is well-known that plays a significant role in regulating tumor metabolic homeostasis, including the Warburg effect, which ultimately facilitates the growth and spread of cancerous cells [73]. The upregulation of circNRIP1 in GC alters glucose metabolism and autophagy via the miR-149-5p/AKT/mTOR axis, promoting tumor metastasis via exosomal communication. Targeting circNRIP1 provides new therapeutic insights for gastric cancer [74]. Notably, circRNAs can also influence the Warburg effect by regulating the expression of transcription factors (TFs). C-myc, one of the crucial TFs that regulates the Warburg effect, is ubiquitinated and degraded by the circ-FBXW7-encoded protein FBXW7-185aa, which provides novel insights regarding the function of circRNAs in glycolysis [75]. In addition, many other exosomal circRNAs, such as exosomal circFBLIM1, exosomal circ-MEMO1, exosomal circ-ZNF652, have been found to influence tumor progression through promotion of glycolysis [76–78]. The Warburg effect also results in the release of exosomal circ_0072083 from drug-resistant cells in gliomas, contributing to increased Temozolomide (TMZ) resistance. It may be a new target for the treatment of TMZ resistance in gliomas [79].

Cholesterol is an essential lipid for cellular function. Abnormalities in cholesterol metabolism such as upregulation of cholesterol synthesis, increased cholesterol uptake and abnormal accumulation of large amounts of metabolites can occur in tumor tissue to promote cancer cell proliferation and metastasis [80, 81]. Squalene epoxidase (SQLE) is a monooxygenase that catalyzes the conversion of squalene to 2,3-epoxy squalene in the cholesterol synthesis pathway. And high expression of SQLE is associated with poor prognosis in breast and hepatocellular carcinoma. Qian et al. have confirmed that circ_0000182, which is highly expressed in gastric adenocarcinoma, can target miR-579-3p and promote SQLE expression, cholesterol synthesis and cell proliferation through a ceRNA mechanism, suggesting the potential application of targeting circRNA in tumor cholesterol metabolism [82]. A novel hypoxia-responsive circINSIG1-encoded protein, circINSIG1-121, which promotes ubiquitin-dependent degradation of INSIG1 to induce cholesterol biosynthesis for CRC proliferation and metastasis, providing a new way to consider the crosstalk between hypoxia and cholesterol metabolism, as well as a promising therapeutic target [83]. In addition, excess fatty acids (FAs) in the body can also stimulate ER stress, inducing hepatocellular damage, liver fibrosis and cirrhosis, and accelerating the development of hepatocellular carcinoma [84]. While combining conventional immune checkpoint inhibitors with mitochondrial fatty acid transporter inhibitors (e.g., etomoxir) can reverse the metabolic reprogramming of DC cells and restore the immune killing effect of T cells, which is an ideal medicine to target abnormal lipid metabolism [85]. Furthermore, it has been revealed that circ-DB is highly expressed in HCC patients with a high percentage of body fat and is associated with poor prognosis, suggesting that circ-DB may promote HCC progression through fatty acid synthesis (FAS), providing a new therapeutic target for targeting tumor lipid metabolism [86].

As an important amino acid metabolism process in the human body, glutamine metabolism plays an important role in a variety of physiological processes such as cholesterol and vitamin synthesis, providing many essential nutrients to our body [63]. SLC1A5 variant is a mitochondrial glutamine transporter for cancer metabolic reprogramming [87]. It has been confirmed that circ_0000069, circ_0000808 and circ_0025033 all positively regulate SLC1A5 and glutamine metabolism through ceRNA mechanisms to promote cancer progression [88–90]. Additionally, Song et al. have found that circ_0067717 can promote CRC progression and glutamine metabolism by upregulating SLC7A5 through sponge miR-497-5p [91]. These are new evidence for the involvement of circRNAs in cancer amino acid metabolism and provide new molecular targets for anti-cancer therapies targeting glutamine metabolism.

Recently, it has been shown that the m6A-modified circRBM33-FMR1 complex promotes prostate cancer (PCa) progression by stabilizing PDHA1 mRNA to activate mitochondrial metabolism. More importantly, depletion of circRBM33 reduces ATP, acetyl CoA levels and the NADH/NAD + ratio, as well as increases the therapeutic sensitivity of androgen receptor signaling inhibitors (ARSIs), including enzalutamide and darolutamide. This suggests the important value of circRNA in regulating ARSI therapy, presenting a potential therapeutic target for PCa [92].

Exosomal circRNAs also regulate tumor metabolism by inducing ferroptosis. Ferroptosis is an iron dependent, non-apoptotic form of cell death, primarily due to an imbalance in the production and degradation of intracellular lipid reactive oxygen species, which is closely associated with cancer progression. Glutathione peroxidase 4 (GPX4) is a major scavenger of lipid peroxidation and is considered as a major regulator of ferroptosis [93]. Zhai et al. reported that inhibition of the circIDE/miR-19b-3p/RBMS1 axis in HCC upregulated GPX4 to reduce ferroptosis and promote tumor progression [94]. As an ferroptosis inducer, erastin acts on the cysteine glutamate reverse transporter (systemXc-) to induce cell death by promoting the accumulation of lipid reactive oxygen species (ROS) and reducing the antioxidant capacity of cells, leading to excessive total cellular lipid ROS [95]. CircRNA-ST6GALNAC6 increases the sensitivity of bladder cancer cells to erastin-induced ferroptosis by regulating the HSPB1/P38 axis, which is important for the development and application of ferroptosis intervention methods [96]. Interestingly, exosomal circ93 is critical in ferroptosis desensitization of LUAD through regulating arachidonic acid (AA), which consequently affects ferroptosis-related plasma membrane peroxidation. It suggests that blocking exosomal circ93 reverses ferroptosis desensitization and provides a new therapeutic target for LUAD [97].

Actually, tumor metabolism is accompanied with the tumor immunity, for instance, the NSCLC exosomal circSHKBP1 targets the miR-1294/PKM2 axis to promote glycolysis and macrophage M2 polarization for tumor progression [98]. In bladder cancer (BCa), the exosomal circTRPS1 regulates the intracellular ROS balance and CD8 + T cell exhaustion via the circTRPS1/miR141-3p/GLS1 axis-mediated glutamine metabolism [99].

In conclusion, exosomal circRNAs are strongly associated with multiple metabolic pathways and provide a favorable microenvironment for cancer cells growth and metastasis. Target exosomal circRNAs can help to reprogram and remodel the TME, making it an important consideration in tumor metabolism research. At the same time, many potential targets have been developed for cancer therapy.

### Tumor proliferation and metastasis

Many exosomal circRNAs are involved in the proliferation and metastasis of multiple types of tumors (Fig. 2 and Table 1). Epithelial-mesenchymal transition (EMT) is a significant process of cellular plasticity that plays a role in tumor development, migration and metastasis. Increasing evidence has shown that exosomal circRNAs have an impact on tumor progression through their ability to regulate miRNAs and activate EMT. For example, the CRC serum exosomal circIFT80 promotes cell growth, proliferation, migration, and invasion by functioning as a ceRNA of miR-1236-3p and increasing the expression of HOXB7 [100]. Additionally, hypoxia-induced exosomal circSETDB1 expression enhances migration, invasion and proliferation of normoxic LUAD cells [101]. The hypoxic tumor-derived exosomal circ-133 promotes aggressive growth and EMT in CRC through the miR-133a/GEF-H1/RhoA axis [102]. Moreover, the exosomal circRNA_PVT1 in cervical cancer promotes metastasis by targeting miR-1286 and enhancing EMT [103]. Similarly, exosomal circ-0004277 in HCC stimulates the malignant phenotype and EMT of peripheral cells, promoting HCC invasion [104]. In addition, exosomal circWHSC1 of ovarian cancer cells can metastasize to peritoneal mesothelial cells and promote peritoneal spread and tumor metastasis [105]. Plasma exosomal circ-PDE8A plays an important role in tumor invasion in pancreatic ductal adenocarcinoma (PDAC) [106].

Lymphatic system plays a vital role in the transportation of fluids, biomolecules and cells between the peripheral tissues and the central circulation through its network of lymphatic vessels and lymph nodes. Lymph node metastasis (LNM) is an important step and is often associated with a poor prognosis [107]. Serum and plasma-derived exosomal hsa_circ_0026611 and hsa_circRNA_0056616 have been shown to be potential biomarkers of tumor lymph node metastasis and poor prognosis [108, 109]. In addition, the exosomal hsa_circ_0000437 has also been found to induce LNM via an HSPA2-ERK signaling pathway, independent of VEGF-C, presenting a novel target for GC therapy (Table 1) [110]. Furthermore, exosomal circ_0026611 has been demonstrated to promote lymph angiogenesis in ESCC by inhibiting PROX1 acetylation and ubiquitination, providing a new perspective on tumor lymph node metastasis [111].

Overall, exosomal circRNAs play a crucial role in regulating tumor proliferation and metastasis by sponging miRNAs and activating EMT [112–115]. And some exosomal circRNAs can promote lymph node metastasis to facilitate tumor progression. Understanding the underlying mechanism of exosomal circRNAs in tumor metastasis is crucial for the development of anti-metastasis therapy.

### Drug resistance

Exosome-mediated intercellular communication has been found to be a novel mechanism of drug resistance in cancer. This phenomenon provides new avenues for exploring therapeutic targets for the treatment of cancer. There is evidence that certain exosomal circRNAs can contribute to drug resistance (Fig. 2 and Table 1). Interestingly, circATG4B-222aa [116], a protein encoded by circATG4B gene, has been shown to increase resistance to the drug oxaliplatin in colorectal cancer by blocking the interaction between TMED10 and ATG4B. Similarly, circ_0000337 has been identified as a potential target for drug resistance in esophageal cancer, as it promotes resistance to the drug cisplatin (CDDP) by interacting with miR-377-3p and JAK2 [117]. While, exosomal circVMP1 promotes CDDP resistance delivery in NSCLC [118].

In glioma, exosomal circWDR62 derived from temozolomide (TMZ)-resistant cells can be delivered to receptor-sensitive cells, conferring TMZ resistance and promoting tumor malignant phenotypes, including proliferation, migration, and invasion [119]. Moreover, exosomal circDNER has been found to increase paclitaxel resistance and tumorigenicity in lung cancer by sponging miR-139-5p, thus promoting ITGB8 expression [120]. Exosomes derived from drug-resistant neuroblastoma cells can deliver circDLGAP4 to drug-sensitive cells, resulting in increased doxorubicin resistance and glycolysis through the inhibition of miR-143 and the promotion of HK2 expression [121]. In addition, exosomal circRNAs regulated by hypoxia have also been shown to contribute to tumor progression and drug resistance. For instance, hypoxia-induced exosomal circZNF91 has been found to enhance glycolysis and resistance to the drug gemcitabine in pancreatic cancer (PC) cells by promoting the stability of HIF-1*α* protein through miR-23b-3p and SIRT1PC [122].

Overall, exosomal circRNAs can induce tumor cell resistance to chemotherapy or radiotherapy and transmit this resistance to sensitive cells. Therefore, these circRNAs could be crucial targets for overcoming drug resistance in cancer.

## Non-tumor-derived exosomal circRNAs in tumor progression

**Fig. 3:**
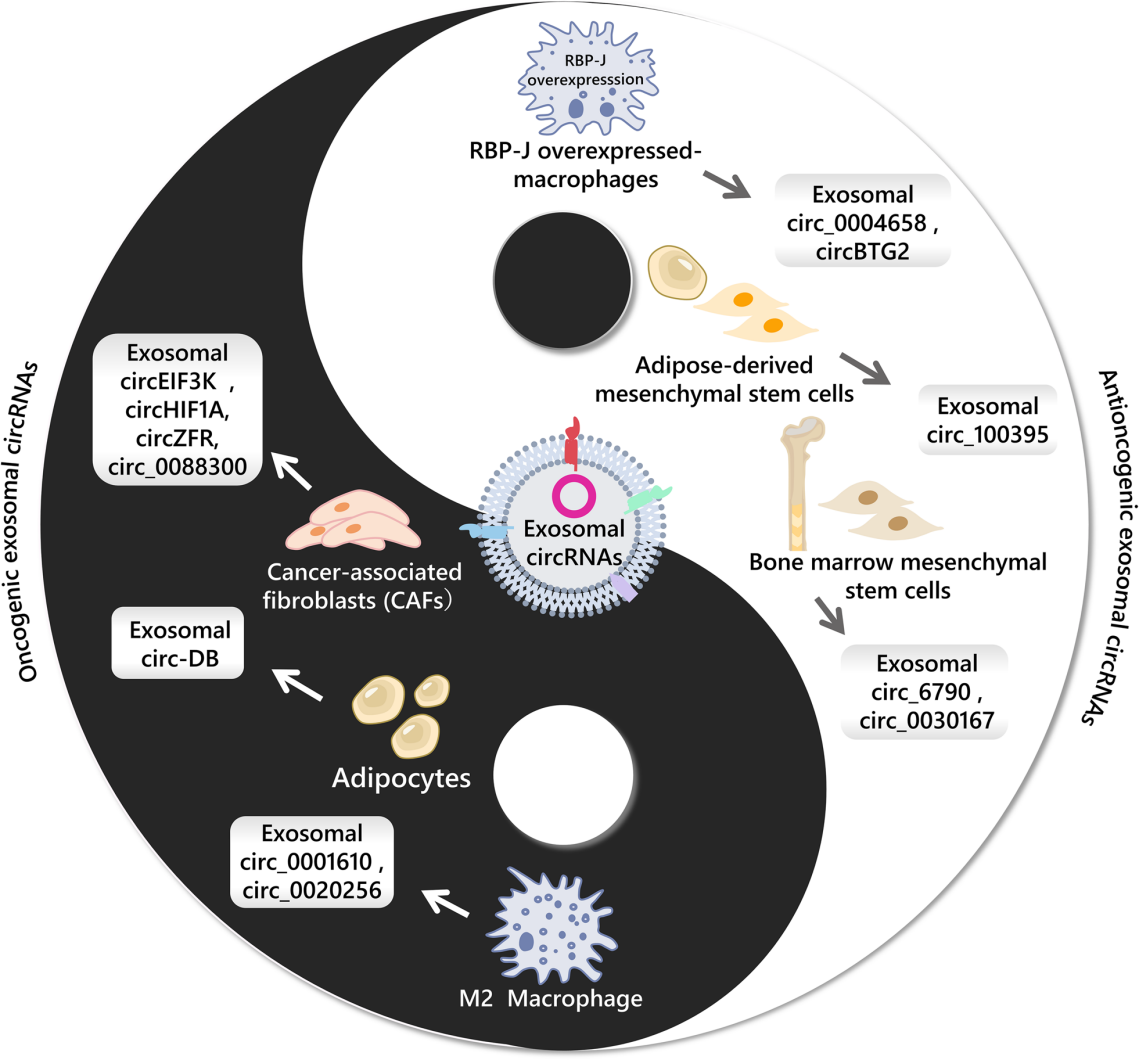
The roles of non-tumor-derived exosomal circRNAs in tumor progression

Cellular components in the tumor microenvironment communicate with tumor cells through exosomes and can impact tumor progression by releasing exosomal circRNAs (Fig. 3 and Table 2).

**Table 2:** Non-tumor-derived exosomal circRNAs in tumor progression

Exosomal circRNA	Parent cell	Target cell	Expression	Signaling pathway	Functions	References
circ_0020256	M2 macrophages	Cholangiocarcinoma cells	↑	circ_0020256/miR-432-5p/E2F3	Promote the proliferation, migration, and invasion of CCA cells	[123]
hsa_circ_0001610	M2 macrophages	Endometrial cancer cells	↑	hsa_circ_0001610/miR-139-5p/cyclin B1	Reduce radiosensitivity of EC cells	[124]
has_circ_0004658	RBPJ-overexpressed macrophages	Hepatocellular carcinoma cells	↓	hsa_circ_0004658/miR-499b-5p/JAM3	Inhibit HCC progression	[127]
circBTG2	RBPJ-overexpressed macrophages	Glioma cells	↑	circBTG2/miR-25-3p/PTEN	Inhibit glioma progression	[128]
circEIF3K	CAFs	Colorectal cancer cells	↑	circEIF3K/miR-214/PD-L1	Promote proliferation, invasion and tube formation of CRC cells	[136]
circHIF1A	CAFs	Breast cancer cells	↑	circHIF1A/miR-580-5p/CD44	Promote breast cancer proliferation and stem cell stemness	[137]
circZFR	CAFs	Hepatocellular carcinoma cells	↑	circZFR/STAT3/NF-κB	Promote HCC growth and CDDP resistance	[138]
circ_0088300	CAFs	Gastric cancer cells	↑	circ_0088300/miR-1305/JAK/STAT	Promote GC cell proliferation, migration and invasion	[14]
circ-DB	Adipocytes	Hepatocellular carcinoma cells	↑	circ-DB/miR-34a/USP7/Cyclin A2	Promote HCC growth and reduce DNA damage	[86]
circ_6790	Bone marrow mesenchymal stem cells	Pancreatic ductal adenocarcinoma cells	↑	circ_6790/CBX7/S100A11	Hamper immune escape in PDAC cells	[141]
circ_0030167	Bone marrow mesenchymal stem cells	Pancreatic cancer	↑	circ_0030167/miR-338-5p/Wif1/Wnt 8/β-catenin	Inhibit the stemness of PC cells and tumor progression	[142]
circ_100395	Adipose-derived mesenchymal stem cells	Non-small cell lung cancer cells	↓	circ_100395/miR-141-3p/Hippo/YAP	Inhibit NSCLC malignant transformation	[143]

### Immune cell-derived exosomal circRNAs in tumor progression

Due to the two phenotypes of anti-tumor (M1) and pro-tumor (M2), exosomal circRNAs released by tumor-associated macrophages (TAM) also play different roles in cancer progression. For example, circ_0020256 in the M2 TAM-secreted exosomes regulates the proliferation, migration and invasion of cholangiocarcinoma cells by targeting the miR-432-5p/E2F3 axis [123]. Similarly, research has shown that hsa_circ_0001610 transferred from the M2 TAMs to endometrial cancer cells by exosomes, and impairs the radiosensitivity of the cancer cells [124]. The recombination signal-binding protein-Jκ (RBP-J) is a transcriptional regulator and a marker of Notch signaling activation [125]. The Notch-RBP-J signaling is associated with polarization of inflammatory macrophage [126]. Exosomal hsa_circ_0004658, produced by RBP-J overexpressing macrophages, has been shown to inhibit liver cancer progression through the miR-499b-5p/JAM3 pathway [127]. Moreover, exosomal circBTG2 from macrophages overexpressing RBP-J targets miR-25-3p/PTEN pathway to hinder glioma progression [128]. Furthermore, an interesting finding highlights that exosomal miR-628-5p from M1 polarized macrophages inhibits the modification of m6A circFUT8 and decelerates the progression of HCC. This reveals a novel mechanism by which immune cell-derived exosomal cargos regulate circRNA and downstream genes in tumor cells and also offers a new potential therapeutic target for HCC therapy [129].

Additionally, although reports regarding immune cell-derived exosomal circRNAs are infrequent, there are already many reports describing the important role of other cargos carried by exosomes such as non-coding RNAs and proteins in cancer progression. For example, miR-765, which is released from CD45RO-CD8 + T cell-derived exosomes, has been found to impede the progression of uterine corpus endometrial cancer (UCEC) via the PLP2/Notch signaling pathway [130]. And NK cell exosomal miR-186 inhibits neuroblastoma growth and impedes immune escape [131]. Furthermore, exosomal cargos derived from neutrophils, DC cells and mast cells, among others, have been demonstrated to influence tumor progression [42, 132, 133].

### Non-immune cell-derived exosomal circRNAs in tumor progression

One key component of the tumor microenvironment is cancer-associated fibroblasts (CAFs), which are widely distributed stromal cells that play a significant role in regulating tumor growth, metastasis, angiogenesis [134], and drug resistance [135]. For example, hypoxia-induced CAFs release exosomal circEIF3K, promoting CRC proliferation, invasion and tube formation through targeting miR-214/PDL1, potentially offering new therapy targets for CRC [136]. Additionally, hypoxic CAF-derived exosomal circHIF1A has been shown to play a role in the stem cell properties of breast cancer, suggesting that circHIF1A may serve as a therapeutic target [137]. Furthermore, research has demonstrated that CAF-derived exosomal circZFR inhibits STAT3/NF-κB pathway in liver cancer cells to promote tumor growth and drug resistance [138]. And CAF-derived circ_0088300, packaged into exosomes driven by KHDRBS3, is delivered to gastric cancer cells to target miR-1305/JAK/STAT and accelerate tumorigenesis [14]. These examples highlight how CAFs can facilitate the development of various tumors by creating a supportive microenvironment and interacting with cancer cells. However, further research is needed to fully understand the interaction between circRNAs and CAFs in influencing tumor progression.

Adipocytes, an important contributor to metabolism, supply various nutrients such as lipids and enzymes, to the tumor microenvironment and support tumor cells [139]. Adipocyte-secreted exosomal circ-DB has been shown to promote liver cancer growth by targeting deubiquitination-related USP7 and reducing DNA damage [86].

Mesenchymal stem cells (MSCs), an important component of the microenvironment, have both tumor-promoting and tumor-suppressive effects [140]. For example, exosomal circ_6790 produced by bone marrow mesenchymal stem cells (BM-MSCs) can be transferred to pancreatic ductal adenocarcinoma, altering DNA methylation of CBX7 and S100A11, and hindering immune escape [141]. Additionally, Yao et al. have confirmed that BM-MSC exosomal circ_0030167 targets miR-338-5p to inhibit the progression and stemness of PC cells, providing a potential target for PC therapy [142]. Moreover, exosomal circ_100395 from adipose-derived mesenchymal stem cells (AMSCs) regulates the Hippo/YAP signaling pathway, increasing LATS2 expression, and suppressing malignant transformation in NSCLC [143].

The delivery of exosomal circRNAs between tumor cells and microenvironmental cells has sparked considerable interest in field of tumor therapy and mechanisms. While current research has largely addressed the influence of tumor cell exosomal circRNAs on microenvironmental cells, we posit that exosomal circRNA derived from non-tumor cell sources can represent promising targets for novel therapeutic interventions in cancer progression.

## Clinical significance

**Fig. 4:**
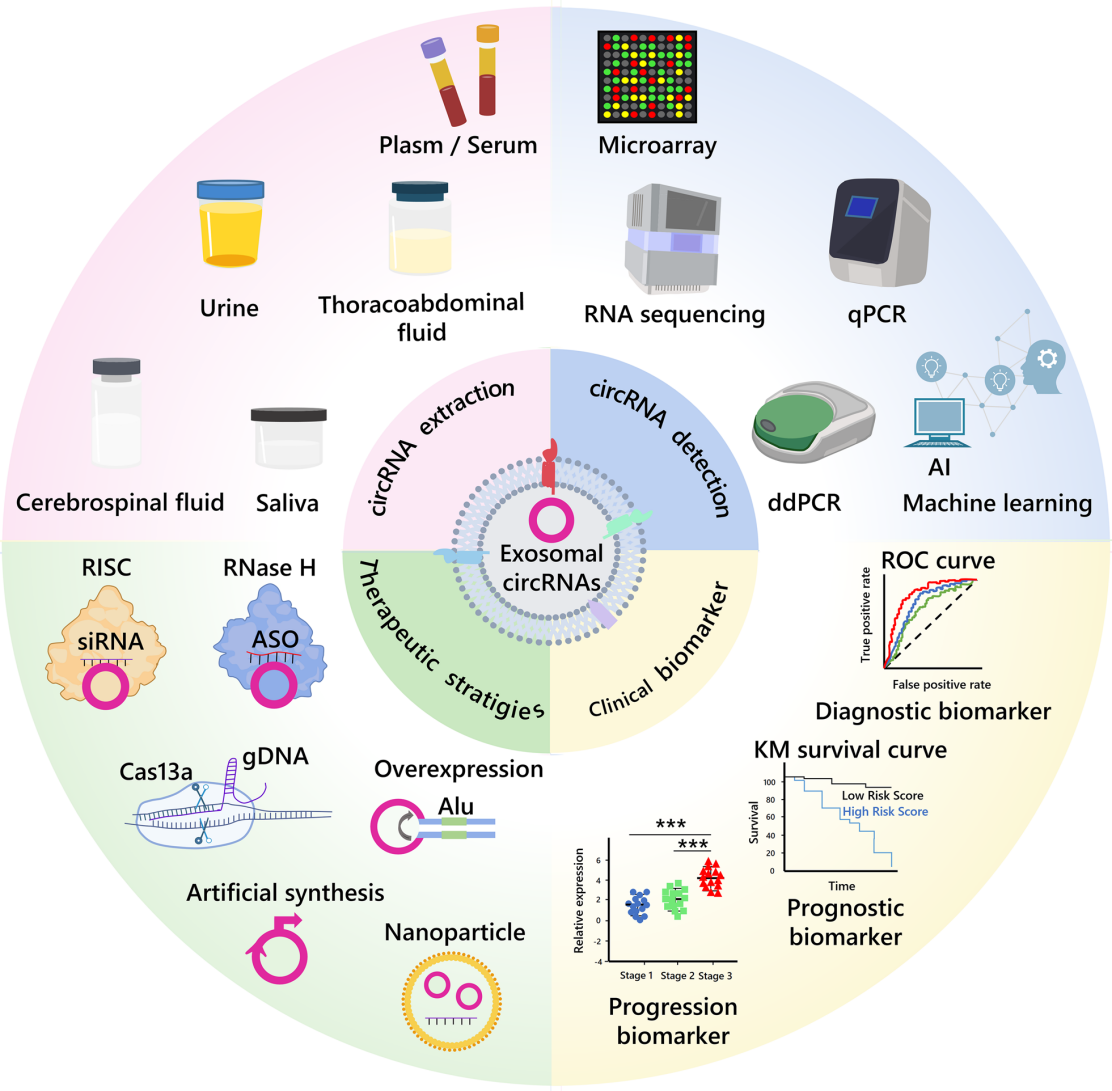
Clinical applications of circRNAs and exosomal circRNAs in tumors, including circRNA extraction, detection, as clinical markers and therapeutic strategies

Exosomal circRNAs offer several advantages, such as high stability, abundance, and long half-life, and they are present in human body fluids such as plasma, serum, and urine [56, 144, 145] (Fig. 4). In addition to their aberrant expression in tumor tissues, exosomal circRNAs have been widely shown to be associated with the progression and clinical features of various cancers. They can serve as early diagnostic and prognostic markers for cancer [146, 147]. Currently, RNA microarrays or sequencing followed by real-time quantitative reverse transcription PCR (qRT-PCR) are commonly used to detect differentially expressed circRNAs, but this can be challenging for exosomal circRNAs with low abundance. To address this issue, droplet digital PCR (ddPCR), which offers high accuracy, sensitivity, reproducibility, and absolute quantification, is increasingly being used to detect exosomal circRNAs in liquid biopsy [148, 149]. In addition, machine learning is being used to improve the accuracy of clinical decisions, particularly when combined with ddPCR. For instance, He et al. have applied a machine learning approach to accurately detect five differential circRNAs in urine extracellular vesicles using ddPCR, which improved the diagnostic efficacy for high-grade prostate cancer [145]. Furthermore, new methods for detecting exosomal circRNAs based on biosensors, electrochemistry, and rolling loop amplification are attracting increasing attention [150–152].

### Exosomal circRNA functions as clinical biomarker

Increasing evidence suggests that exosomal circRNAs hold great promise as diagnostic and prognostic biomarkers for various types of tumors (Fig. 4 and Table 3) [153, 154]. For instance, a combination of exosomal hsa_circ_0001439, hsa_circ_0001492, and hsa_circ_0000896 demonstrated better diagnostic sensitivity and specificity for LUAD, with an AUC value of 0.805. Notably, the diagnostic sensitivity (73.2%, 66.1%,62.2%) and specificity (70.8%, 84%, 87.5%) of the three serum exosomal circRNAs were higher than those of serum circRNAs [155]. Hsa_circ_0055202, hsa_circ_0074920 and hsa_circ_0043722 have been confirmed to highly expressed in plasma exosomes of GBM patients by circRNA microarray technology. The area under the curve (AUC) of the triple combination is high at 0.988 and 0.925 in the training and validation cohorts, respectively [156]. Also, a recent study has shown that high expression of circMYC in serum exosomes of patients with nasopharyngeal carcinoma (NPC) is associated with tumor size, lymph node metastasis, TNM stage, survival rate, and disease recurrence, with an AUC of 0.945, granting a new biomarker for the diagnosis and prognosis of NPC [157]. Interestingly, machine learning algorithms have been used to construct a nine-circRNA combination classifier with an SVM model (BCExoC) to improve the diagnostic value for breast cancer, with AUCs of 0.83 and 0.80 in the training and validation cohorts, respectively [158]. These findings suggest that combining biomarkers with machine learning algorithms may be a crucial technique in future advancements to improve diagnostic efficacy.

**Table 3:** Exosomal circRNAs as cancer biomarkers

Exosomal circRNA	Type of disease	Source of exosome	Expression	Clinical relevance	Case number	AUC	Sensitivity and specificity	References
circPDLIM5, etc.	Prostate cancer	Urine	↑	_	263 in training cohort; 497 in validation cohort 1; 505 in validation cohort 2	0.820 in training cohort; 0.807 in validation cohort 1; 0.810 in validation cohort 2	_	[145]
circ_0047921, circ_0056285, circ_0007761	Non-small cell lung cancer	Serum	↓ ↓ ↑	circ_0056285: clinical stages and lymph node metastasis	120 in training set; 62 in validation set1; 63 in validation set2	Combined:0.926 in training set;0.919 in validation set	_	[153]
circ_0070396	Hepatocellular cancer	Plasma	↑	AFP, AST, ALT, ALB, TBIL, DBIL	111	0.8574	Sensitivity:62.16% Specificity:98.15%	[154]
hsa_circ_0001439, hsa_circ_0001492, hsa_circ_0000896	Lung adenocarcinoma	Serum	↑ ↑ ↑	TNM stage, tumor size, NSE, CEA	134	Combined:0.805	Sensitivity:73.2%; 66.1%;62.2% Specificity:70.8%;84%;87.5%	[155]
hsa_circ_0055202, hsa_circ_0074920, hsa_circ_0043722	Glioblastoma multiforme	Plasma	↑ ↑ ↑	_	120	Combined:0.988 in training set; 0.925 in validation set	_	[156]
circMYC	Radio-resistant nasopharyngeal carcinoma	Serum	↑	Tumor size, lymph node metastasis, TNM stage, survival rate, and disease recurrence	210	0.945	_	[157]
BCExoC (hsa_circ_0002190, etc.)	Breast cancer	Plasma	↑	_	144 in training cohort; 101 in testing cohort;	SVM:0.83 in training cohort; 0.80 in testing cohort	_	[158]

However, to validate the clinical utility of exosomal circRNA biomarkers, more rigorous data and a large number of clinical samples are needed (Table 3). Furthermore, since biomarkers with high specificity and sensitivity are lacking, a combination of exosomal circRNAs with traditional biomarkers such as CEA and CA19-9 may yield better diagnostic outcomes. After large-scale sample collections, clinical cohort studies, and multi-center collaborative research, exosomal circRNAs may be considered promising diagnostic markers.

Given the potential value of exosomal circRNAs in tumor diagnosis and prognosis, there have been a variety of clinical trials targeting exosomal circRNAs as tumor biomarkers. Details of current data from both domestic and international clinical trials of circRNAs and exosomal circRNAs as diagnostic or prognostic biomarkers for cancer, including a variety of cancers such as cholangiocarcinoma, pancreatic cancer and prostate cancer, are summarized in Table 4. (data from https://www.chictr.org.cn/ and https://clinicaltrials.gov/) In addition, as emerging clinical biomarkers, most of the current clinical trials are in Phase 0 and some projects have not yet initiated recruitment of recipients. However, we believe that with the support of more and more research data, exosomal circRNAs will become very promising and reliable tumor biomarkers and will eventually go to the clinic.

**Table 4:** Clinical trials with circRNAs and exosomal circRNAs as cancer diagnosis or prognosis biomarkers

Registration number/NCT number	Study type	Study phase	Recruiting status	Tumor type	Sample name	Sample size
ChiCTR2300069863	Observational study	0	Recruiting	Cholangiocarcinoma	Bile, serum	320
ChiCTR1900027419	Basic science	0	Not yet recruiting	Lung cancer	Blood	58
ChiCTR1900024188	Diagnostic test	0	Recruiting	Prostate cancer	Urine	300
ChiCTR1800019529	Diagnostic test	Diagnostic new technique clinical study	Recruiting	Prostate cancer	Plasma	200
ChiCTR1800018038	Diagnostic test	Diagnostic new technique clinical study	Not yet recruiting	Pancreatic cancer	Blood	20
NCT05771337	Observational study	Not available	Not yet recruiting	Breast cancer	Blood	80
NCT04584996	Observational study	Not available	Recruiting	Pancreatic cancer, biliary tract cancer	Blood, bile	186
NCT04464122	Observational study	Not available	Recruiting	Neuroendocrine neoplasm	Not available	60
NCT03334708	Observational study	Not available	Recruiting	Pancreatic cancer	Blood	700

### Therapeutic strategies based on exosomal circRNA

Exosomal circRNAs have been implicated in cancer, and targeting them for modulation has led to the development of novel therapeutic strategies for cancer. Here, we describe the therapeutic strategies and limitations of direct targeting of circRNAs as well as present the current status and prospects for nanoparticles and exosome-based delivery of circRNAs (Fig. 4).

#### Strategies for targeting circRNAs

Technologies such as small interfering RNA (siRNA), antisense oligonucleotide (ASO), and CRISPR cas13a have been designed to target oncogenic circRNAs. For example, in vivo treatment with circNRIP1 siRNA in Patient-derived tumor xenograft (PDX) mice significantly reduced tumor weight and volume [74]. Additionally, ASOs targeting circRHOBTB3 inhibited CRC growth and metastasis in vitro and in vivo [159]. CRISPR/Cas13a-mediated knockdown of hsa_circ_0000190(C190) inhibited NSCLC cell proliferation and migration in vitro and reduced tumor growth in vivo [160]. However, techniques based on silencing circRNAs, such as siRNA and ASO, usually inevitably cause off-target effects, resulting in inconsistent efficiency in silencing circRNAs. In addition, siRNAs with a high G/C base content may be less effective. As a Cas13 effector, the PspCas13b effector shows a higher specificity for Drosophila circRNA, but still produces off-target effects [161].

Conversely, overexpression vectors have been used to promote the production of tumor-suppressor circRNAs such as circFNDC3B, which inhibits angiogenesis and CRC progression [12, 162]. Notably, due to the specificity of the loop structure, there may be incorrect splicing or insertion and deletion of bases in the construction of circRNA overexpression vectors to cause failure or inefficient loop formation. The incorporation of a circularized expression framework into the circRNA vector is important for overexpression of circRNA. In addition, circRNA primers used for qPCR experiments must specifically and efficiently to amplify circRNAs rather than other transcripts in order to properly validate circRNA silencing and overexpression efficiency.

Intriguingly, a growing number of studies have confirmed that circRNAs can encode functional proteins and participate in cancer progression [163]. In order to increase the value of these circRNAs, researchers are working to synthesize circRNAs artificially to avoid the limitations of linear mRNAs. For example, Alex Wesselhoeft's team used an engineering approach to prepare a circRNA with the potential to efficiently encode proteins for efficient and stable expression in eukaryotic cells [164]. Excitingly, through optimizing circRNA translation conditions, Chen et al. have improved the yield of circRNA-translated proteins to hundreds of folds, enabling more sustainable expression of translated protein products for biological functions in vitro and in vivo, which holds tremendous promise for application [165]. A recent study reported that down-regulated expression of circ-INSR may be an important target in the process of cardiotoxicity and cardiac remodeling. The use of adeno-associated virus (AAV) and artificially prepared circ-INSR mimics prevents and reverses adriamycin-mediated cardiomyocyte death and improves cardiac function. It is significant for engineering circRNAs from basic to clinical [166]. Controversially, engineered circRNAs produced exogenously have been demonstrated to induce activation of the immune system in vivo [167, 168]. It is also a limitation of engineered circRNA. Through comparing the efficiency and immunogenicity of circRNAs prepared by various in vitro synthesis methods, Chen et al. have found that circRNAs synthesized by T4 RNA ligase have lower immunogenicity, which is an important foundation for the engineering of circRNAs in gene therapy [169]. Furthermore, the generation, purification, primary sequence, secondary structure and RNA modification of engineered circRNAs are all important factors affecting their immunogenicity [170]. A strategy of encapsulating synthetic circRNAs with RNA-binding proteins and splicing factors may be an effective means of suppressing innate immune activation [171].

#### Therapeutic strategies using nanoparticle-delivered circRNAs

While most studies have used adenoviral and lentiviral vectors as viral delivery systems to target circRNAs, there are high costs and potential biosafety issues, as well as limited ability to carry genes. Nanoparticles are materials in the size range of 10 to 1000 nm, which have the advantages of low cost, ease for preparation, good biocompatibility, biodegradability and high safety compared to viral vectors, and play an important role in bioimaging, molecular detection, disease diagnosis and drug delivery [172, 173]. A variety of organic materials (such as phospholipid, polymers, etc.) and inorganic materials (such as gold and metal oxides.) can be used to produce nanoparticles to protect RNA from degradation [174]. Emerging evidence suggests that nanoparticle-based delivery systems for circRNAs may also be a promising therapeutic strategy. A lipid nanoparticle (LNP) system developed for in vitro and in vivo delivery of circRNA triggered adaptive immune activation and showed excellent antitumor efficacy in multiple mouse tumor models, offering new prospects for the development of cancer RNA vaccines [175]. Qu et al. have generated high concentrations and abundance of circRNA-RBD with antigenic potential encoding SARS-CoV-2-RBD in vitro using type I intron nuclease and T4 RNA ligase and have demonstrated that SARS-CoV-2 circRNA-RBD vaccine induces sustained humoral immune responses in mice and rhesus monkeys with LNP delivery system. It shows higher and more sustained antigen expression than the mRNA vaccine. In addition, the circRNA vaccine does not cause clinical signs of disease in rhesus monkeys and exhibits a good biosafety [176]. In addition, Du et al. have demonstrated that delivery of circ-Foxo3 plasmids coupled with gold nanoparticles in vivo can target Foxo3 and upregulate PUMA to induce apoptosis and enhance survival in mice. Nevertheless, more clinical trials are needed to evaluate its biosafety [177]. It is also possible to use nanoparticles to encapsulate siRNA to improve delivery efficiency. In the orthotopic tumor model and lung metastasis model mice, the poly lactic-co-glycolic acid (PLGA)-based si-cSERPINE2 nanoparticles effectively attenuated breast cancer progression without apparent systemic toxicity, hepatotoxicity and renotoxicity [37]. Meng et al. used nanoparticles made from PLGA and polyethylene glycol (PEG) chains to deliver si-circROBO1 in vitro and in vivo with good anticancer effects and no significant organ toxicity [178].

Nevertheless, we still need to develop more novel drug delivery systems with precision drug delivery capabilities to facilitate circRNAs clinical translation. As an advanced delivery system, the stimulus-responsive delivery system protects the precise delivery of RNA with dynamic activity compared to conventional non-stimulus-responsive nanocarriers. Some popular stimulus-responsive nanomaterials, including pH-responsive nanocarriers, enzyme-responsive nanocarriers, hypoxia-responsive nanomaterials, etc., are more suitable for tumor sites [179]. You et al. have reported a circ_0058051-siRNA delivery system based on superparamagnetic iron oxide nanoparticles (SPIONs) to protect it from degradation in serum and ultimately inhibit HCC progression in vivo with a good biosafety [180]. Stimulus-responsive nanoparticle-based systems are now widely used to deliver siRNA, miRNA, etc. [181, 182]. We believe that its application to the delivery of circRNAs will become a future research trend.

#### Therapeutic strategies using exosome-delivered circRNAs

Exosomes are 30–150 nm extracellular vesicles with an average size of 100 nm that have good stability, small size, and low toxicity, making them ideal drug delivery carriers [6]. Compared to synthetic carriers such as liposomes and nanoparticles, exosomes have attracted much attention in recent years because of their strong biocompatibility, low immunogenicity and high chemical stability [183, 184].

Engineered exosome-based therapies have been developed for various diseases, including cancer [185, 186]. Therapeutic strategies based on exosomal circRNAs are particularly promising. Wang et al. have confirmed that exosomal ciRS-122 promotes glycolysis and induces chemical resistance through the miR-122/PKM2 axis in CRC. Both in vivo and in vitro experiments have demonstrated that si-ciRS-122 delivered by exosomes reversed oxaliplatin resistance and glycolysis and inhibited tumor proliferation in CRC [71]. RGD-exo-circDIDO1 inhibited the development of GC and was found to have no potential toxic effects [187]. Engineered RVG-circSCMH1-EVs selectively delivered circSCMH1 to the brain, enhancing neuronal plasticity in mice and monkeys after stroke [188]. However, packaging and delivering large circRNAs into exosomes is challenging, and there is a need to develop more effective approaches.

Currently, a variety of exosome-delivered vaccines have entered clinical trials. For example, a trial of vaccination with dendritic cell-derived exosomes loaded with tumor antigens has recruited 41 participants and is currently in phase II clinical trials (NCT01159288). And another open dose-escalation clinical study of chimeric exosomal tumor vaccines for recurrent or metastatic bladder cancer is still recruiting and is currently in an early phase I clinical trial (NCT05559177). Some relevant clinical trials have also been registered in China (ChiCTR2100050939, ChiCTR2000034241). Although there are no clinical trials available, we believe that circRNA-based exosome delivery technology will shine in the field of cancer therapy in the future.

The last but not least, the delivery of drugs based on plant or food exosomes is a promising trend. They have the advantages of wider availability, lower cost and ease to access compared to cellular exosomes which are already used in a variety of diseases including cancer [189–191]. Currently, grape extract (NCT01668849), ginger and aloe exosomes (NCT03493984) have been approved for clinical trials. This is certainly the future direction of engineered exosomes.

Taken together, exosomal circRNAs-based therapeutic approaches hold great promise, and more research is needed to explore their potential as a therapeutic strategy for cancer. In addition, exosomal circRNA delivery technologies based on nanoparticles and plant exosomes are promising stars for tomorrow.

## Conclusions and perspectives

Exosomal circRNAs have become a focal point of research due to their involvement in cancer progression, including immune escape, angiogenesis, metabolism, drug resistance, proliferation and metastasis. Both tumor and non-tumor derived extracellular circRNAs play a double-edged sword or “pro-and anti-cancer” role in cancer progression through mechanisms such as miRNA sponges, interactions with target proteins, protein scaffolds, translation proteins and small molecule peptides, indicating that these molecules have been providing insights into cancer pathogenesis and treatment. Moreover, increasing evidence suggests that extracellular circRNAs have the potential to become biomarkers for cancer diagnosis and prognosis, especially when combined with traditional tumor markers, which significantly improves the sensitivity and specificity of diagnosis and efficacy analysis. At the same time, the application of machine learning and artificial intelligence can establish a multi-molecule diagnosis panel system and platform based on extracellular circRNAs, providing ideal evidence for precise diagnosis and treatment of cancers. Exosomal circRNAs present amazing bidirectional regulating ability to the remodeling immunity microenvironment no matter in tumor cells or immune cells. And exosomal circRNAs involve in the reprogramming tumor metabolism such as glycolysis, lipid metabolism, amino acid metabolism, oxidative respiration through diverse enzymes, TFs, and signaling pathways. Especially, exosomal circRNAs can alter the condition of immune cell, immune checkpoint molecules and metabolism simultaneously. These pave a way for precise dynamic surveillance and target treatment.

However, the specific mechanisms by which components in the tumor microenvironment (TME) affect tumor cell fate through exosomal circRNAs remain unclear, and further investigation is needed to understand the unique functions of TME components. Additionally, several limitations remain about the use of exosomal circRNAs in body fluids as biomarkers for cancer diagnosis and prognosis, including a limited sample size, insufficient specimens representing various clinical stages and.differential diagnoses of cancer, as well as a lack of standardized and uniformly applied quality control evaluation systems. The engineering strategies for exosomal circRNAs also face limitations such as the size of circRNAs for effective packaging and delivery, as well as safety, pharmacokinetics, and pharmacodynamics considerations. In order to effectively target oncogenic circRNAs, it is imperative to develop knockdown or knockout strategies tailored to a specific circRNA. However, it is inevitable that off-target effects will occur. Furthermore, constructing overexpression vectors for cancer suppressor circRNAs may a challenge due to the specificity of the loop structure of circRNAs which may result in failed loop formation or inefficiency. Artificial preparation of circRNAs presents a promising approach to developing therapeutics; however, the issues of immunogenicity and standardization must be addressed. Another important direction is the engineering of circRNAs using exosomes from immune cells, which have potent anti-tumor activity. Incorporating stimulus-responsive nanocarriers with exosomes may enhance circRNA delivery, especially when adapted to the tumor microenvironment. In addition, circRNA delivery based on plant or food exosomes is a promising strategy due to its easy availability and low cost.

Technological advancements in the extraction and purification of exosomes are necessary to differentiate them from microvesicles, and to ensure their purity and specificity. Innovation in this field, such as the utilization of machine learning, droplet digital PCR, and artificial intelligence, may enhance our ability to identify the involvement of exosomal circRNAs in tumor diagnosis, ultimately leading to better clinical decision-making. Moreover, exosomal circRNAs have significant therapeutic potential, and manipulating exosomes presents new possibilities in the realm of cancer therapy. In conclusion, increasing evidence supports the promising clinical applications of exosomal circRNAs as both novel potential biomarkers and therapeutic targets.

## Data Availability

Not applicable.

## Abbreviations

CircRNA: Circular RNA
EVs: Extracellular vesicles
TME: Tumor microenvironment
MVBs: Multivesicular bodies
DC cell: Dendritic cell
T cell: T lymphocyte
HCC: Hepatocellular carcinoma
PD1: Programmed cell death protein 1
NSCLC: Non-small cell lung cancer
IFN-*γ*: Interferon-*γ*
TNF-*α*: Tumor necrosis factor *α*
MiRNA: MicroRNA
Tregs: Regulatory T cells
PDL1: Programmed cell death ligand 1
LUAD: Lung adenocarcinoma
RCC: Renal cell carcinoma
TAMs: Tumor-associated macrophages
BC: Breast cancer
ER: Endoplasmic reticulum
ESCC: Esophageal squamous cell carcinoma
HuR: Human antigen R
GC: Gastric cancer
IL-6: Interleukin-6
NK cells: Nature killer cells
TAN: Tumor-associated neutrophil
CRC: Colorectal cancer
CTLA4: Cytotoxic T lymphocyte associate protein-4
TIM-3: T-cell immunoglobulin and mucin domain 3
VEGF: Vascular endothelial growth factor
HUVEC: Human umbilical vein endothelial cell
hBMECs: Human brain microvascular endothelial cells
GBM: Glioblastoma multiforme
HK2: Hexokinase 2
2-DG: 2-Deoxy-D-glucose
PKM2: Pyruvate kinase type M2
PEP: Phosphoenolpyruvate
LDHA: Lactate dehydrogenase A
TF: Transcription factor
TMZ: Temozolomide
SQLE: Squalene epoxidase
FAs: Fatty acids
FAS: Fatty acid synthesis
PCa: Prostate cancer
ARSI: Androgen receptor signaling inhibitor
GPX4: Glutathione peroxidase 4
ROS: Reactive oxygen species
AA: Arachidonic acid
BCa: Bladder cancer
EMT: Epithelial-mesenchymal transition
PDAC: Pancreatic ductal adenocarcinoma
LNM: Lymph node metastasis
CDDP: Cisplatin
PC: Pancreatic cancer
HIF: Hypoxia-inducible factor
RBP-J: Recombination signal-binding protein-Jκ
UCEC: Uterine corpus endometrial cancer
CAFs: Cancer-associated fibroblasts
MSCs: Mesenchymal stem cells
BM-MSCs: Bone marrow mesenchymal stem cells
AMSCs: Adipose-derived mesenchymal stem cells
qRT-PCR: Quantitative reverse transcription polymerase chain reaction
ddPCR: Droplet digital polymerase chain reaction
AUC: Area under the curve
NPC: Nasopharyngeal carcinoma
CEA: Carcinoembryonic antigen
CA19-9: Carbohydrate antigen19-9
siRNA: Small interfering RNA
ASO: Antisense oligonucleotide
CRISPR: Clustered regularly interspaced short palindromic repeats
Cas13a: CRISPR-associated protein 13a
PDX: Patient-derived tumor xenograft
AAV: Adeno-associated virus
LNP: Lipid nanoparticle
PLGA: Poly lactic-*co*-glycolic acid
PEG: Polyethylene glycol
SPIONs: Superparamagnetic iron oxide nanoparticles
RVG: Rabies virus glycoprotein
